# Progress in mesenchymal stem cell–based therapy for acute liver failure

**DOI:** 10.1186/s13287-018-0972-4

**Published:** 2018-08-24

**Authors:** Yong-Hong Wang, Dong-Bo Wu, Bing Chen, En-Qiang Chen, Hong Tang

**Affiliations:** Center of Infectious Diseases, West China Hospital of Sichuan University, Chengdu, 610041 China

**Keywords:** Mesenchymal stem cells, Acute liver failure, Treatment

## Abstract

Acute liver failure is a life-threatening clinical syndrome characterized by rapid development of hepatocellular necrosis leading to high mortality and resource costs. Numerous treatment strategies for acute liver failure simply prevent complications and decelerate disease progression. The only curative treatment for acute liver failure is liver transplantation, but there are many restrictions on the application of liver transplantation. In recent years, a growing number of studies have shown that stem cells can effectively treat acute liver failure. Several types of stem cells have been used to study liver diseases; mesenchymal stem cells are most commonly used because they are easy to obtain and present no ethical problems. The aims of this article are to review the current knowledge regarding therapeutic mechanisms of mesenchymal stem cells in acute liver failure, to discuss recent advancements in preclinical and clinical studies in the treatment of mesenchymal stem cells, and to summarize the methodological improvement of mesenchymal stem cell transplantation in treating liver failure.

## Background

Acute liver failure (ALF) is a life-threatening clinical syndrome characterized by rapid hepatocellular necrosis due to various acute injuries induced by hepatotoxic drugs, immune-mediated attack, or viral infections. Notwithstanding the low morbidity of ALF, approximately one and six cases per million individuals annually worldwide, this serious disease will lead to high mortality and resource costs [1, 2]. Currently, many ALF treatment strategies are aimed at simply preventing complications and decelerating disease progression. The only curative treatment for ALF is liver transplantation, but there are many restrictions on the application of liver transplantation because of financial considerations, a shortage of donor livers, and immunosuppression-related complications [3]. Therefore, novel therapeutic methods for patients with ALF are urgently required. In recent years, a growing number of studies have shown that stem cells can effectively treat ALF.

Several types of stem cells, including embryonic stem cells, mesenchymal stem cells (MSCs), induced pluripotent stem cells, hepatic progenitor cells, and hematopoietic stem cells, have been used to study liver diseases [4]; however, MSCs are most commonly used because they are easy to obtain and present no ethical problems [5]. MSCs have the capacity of multiple differentiations and self-renewal and have been proven to be able to differentiate into a series of cell lineages, such as adipocytes, osteoblasts, chondroblasts, and hepatocyte-like cells (HLCs). In addition, they have other properties, including anti-inflammatory effects, anti-apoptosis, immunosuppression, angiogenesis, promotion of tissue repair, and growth factor production. Although there is a great deal of preclinical and clinical research about MSCs in the treatment of ALF, the exact mechanism underlying the therapeutic role of MSCs remains unclear [6]. The aims of this review are to review the current knowledge regarding therapeutic mechanisms of MSCs in ALF, to discuss recent advancements in preclinical and clinical studies in the treatment of MSCs, and to summarize the method improvement of MSC transplantation in treating liver failure.

### MSCs differentiate into hepatocyte-like cells

The idea of using hepatocytes to treat liver failure depends on a simple hypothesis that liver function can be improved by supplementing exogenous hepatocytes. Although liver failure can be treated via hepatocyte transplantation, it also faces multiple problems comprising the shortage of high-quality hepatocytes sources, rejection of allogeneic transplants, difficulty to expand, and losing hepatic characteristics *in vitro* [7, 8]. Previous studies have proven that rodent and human MSCs can differentiate into HLCs *in vitro* and *in vivo*, which is one of the mechanisms of MSCs in the treatment of liver failure. Therefore, MSCs may help resolve issues restricting the application of hepatocyte transplantation. Different groups have established several protocols to induce the differentiation of MSCs into HLCs *in vitro*. MSCs, such as bone marrow (BM-MSCs), adipose tissue (AT-MSCs), umbilical cord (UC-MSCs), and amniotic fluid (AF-MSCs), have been shown to differentiate into HLCs [9–12]. Human BM-MSCs are induced into HLCs via a serum-free maintenance medium of human hepatocyte supplemented with hepatocyte growth factor (HGF) and epidermal growth factor (EGF), which has been proven to retain the qualities of hepatocytes after their regional integration in mouse livers [9]. Differentiation of BM-MSCs and UC-MSCs was induced by differentiation medium supplemented with HGF, basic fibroblast growth factor (bFGF), and nicotinamide, followed by treatment with maturation medium containing dexamethasone, oncostatin M (OSM), and insulin-transferrin-selenium (ITS) [10]. AT-MSCs can differentiate into functional HLCs *in vitro* via culturing in a hepatic culture medium containing EGF, HGF, FGF1, FGF4, ITS, OSM, and dexamethasone. Meanwhile, AT-MSC–derived hepatocytes can be incorporated into the host liver and improve liver functions [11]. AF-MSCs were serum-deprived for 2 d in culture medium supplemented with EGF and bFGF. Differentiation was induced by treating AF-MSCs with differentiation medium containing HGF, bFGF, and dimethyl sulfoxide (DMSO) for 7 d, followed by maturation medium comprising OSM, dexamethasone, and ITS for 2 weeks [12]. Demethylation reagents such as 5-azacytidine have been proven to be useful in inducing MSCs to differentiate into HLCs [9, 13, 14]. Although 5-azacytidine has been applied in clinical treatment of hematologic diseases [15], the side effects, including thrombocytopenia, myelosuppression, and pneumonia, are obvious [16]. Therefore, we should pay attention to its side effects when we use demethylation agents to induce differentiation. HLCs differentiated from different MSC types can be identified via various methods, including observation of hepatocyte-specific morphology, expression of hepatocyte-specific marker genes, and the functions of hepatocytes comprising glycogen storage, albumin production, uptake of low-density lipoprotein, indocyanine green uptake assay, urea secretion, and cytochrome P450 activity.

Many studies have shown that transplanted MSCs can directly differentiate into HLCs *in vivo*. Human UC-MSCs entered the injured liver induced by D-galactosamine/lipopolysaccharide (D-GalN/LPS) in mice and differentiated into HLCs, identified via positive staining of albumin (ALB), alpha fetoprotein (AFP), and cytokeratin 18 (CK18) [17, 18]. The transplantation of human UC-MSCs significantly improves the survival rate of acute hepatic necrosis rats induced by carbon tetrachloride (CCl_4_). The underlying mechanisms may involve human UC-MSC trans-differentiation into HLCs and targeted migration to liver injury sites [19, 20]. However, some experiments have suggested that MSCs cannot directly differentiate into HLCs *in vivo*. Xiao et al. showed that transplanted MSCs have no obvious evidence of hepatocyte trans-differentiation, upon assessing the survival, distribution, and hepatocyte markers of MSCs *in vivo* [21]. Chen et al. proved that AT-MSCs did not differentiate into hepatocytes after engrafting to livers within 3 d [22].

At present, treatment alternatives for liver failure between undifferentiated MSCs and HLCs are still controversial. Zagoura et al. reported that the effect of undifferentiated MSCs is better than that of HLCs, showing that AF-MSC-derived HLCs, compared with AF-MSCs and hepatic progenitor-like cells, failed to enter the damaged liver and contribute to recovery [12]. Similar results were obtained by Wang et al., who observed that HLCs expressed lower levels of HGF and were accompanied by impaired immunosuppression compared with MSCs. Therefore, undifferentiated MSCs may be more suitable than HLCs to treat liver diseases [23]. However, several recent studies have shown that the treatment effects of undifferentiated MSCs and HLCs are similar in ALF [24–27]. Undifferentiated MSCs and HLCs from adipose tissue, bone marrow, and the umbilical cord transplanted in a mouse model of acute fulminant hepatitis were equally able to regenerate injured liver tissue and save almost all of the mice [24, 26]. Similarly, Li et al. found that uninduced BM-MSCs and HLCs had similar effects on the treatment of ALF in rats. Levels of alanine transaminase (ALT), aspartate transaminase (AST), and total bilirubin (TBIL) in the transplantation group were significantly higher than those in the control group and decreased significantly 7 d after transplantation [25]. No studies have reported better therapeutic effects of HLCs than undifferentiated MSCs, based on literature reviews. Therefore, hepatocyte-like differentiation may not be necessary for MSCs to treat liver failure.

### Mechanisms of MSC-mediated immunomodulation

Most previous studies have shown that the therapeutic effects of MSCs in liver failure are potentially based on its release of trophic and immunomodulatory factors. Although the immunomodulatory mechanism of MSCs remains to be elucidated, they are likely to regulate immune cells by secreting soluble factors and intercellular contacts. MSCs can regulate adaptive and innate immune responses by inhibiting T cells and dendritic cells, reducing the activation and proliferation of B cells, promoting the production of regulatory T (Treg) cells, and inhibiting the proliferation and cytotoxicity of natural killer (NK) cells [28–33]. When MSCs play an immunoregulatory role, transforming growth factor-beta (TGF-β) and interleukin 10 (IL-10) are key factors regulating numerous inflammatory cells. Fang et al. showed that the levels of TGF-β and IL-10 in serum increased significantly after injecting UC-MSCs but that the levels of IL-6, tumor necrosis factor-alpha (TNF-α), and CD8^+^ T cells in peripheral blood decreased significantly, which resulted in the repair of liver injury and improved disease developing and mortality rates [29]. Meanwhile, BM-MSCs can induce transient T-cell apoptosis through the Fas ligand (FasL)-dependent Fas pathway, and apoptotic T cells subsequently trigger macrophages to produce high levels of TGF-β, which leads to the upregulation of Treg cells to induce immune tolerance [28]. In fulminant hepatic failure (FHF), the therapeutic effects of MSCs are achieved primarily by reducing hepatic CD4^+^ T-cell infiltration and activation, inhibiting T helper 1 (Th1) cells, and inducing Treg cells. Moreover, MSCs can induce a distinct liver population of CD11c^+^MHCII^hi^CD80^lo^CD86^lo^ regulatory dendritic cells that induced Treg cell differentiation through TGF-β production [30]. It has been reported that BM-MSC infusion could improve the immunoregulatory activity by inhibiting liver NKT cells and this inhibition is systemic, not limited to the liver [33]. MSCs can inhibit cytotoxic CD8^+^ T lymphocyte (CTL) and NK cells through intercellular contact and paracrine factors such as indoleamine 2,3-dioxygenase (IDO), TGF-β, and prostaglandin E_2_ (PGE_2_) [3]. Of note, TGF-β is a two-edged sword with immunosuppressive effects to alleviate liver inflammation [3, 28, 30] but can also promote the progression of liver fibrosis [34, 35]. The immunomodulatory effect of MSCs on transforming the body into an anti-inflammatory state is achieved by upregulating anti-inflammatory Treg cells and reducing Th1 and Th17 cells in FHF. In addition, establishment of the anti-inflammatory state after MSC transplantation may be indirectly induced via upregulation of M2-type macrophages, which secrete various anti-inflammatory factors, including chemokine ligand 1 (CCL-1) and IL-10, which upregulate Th2 and Treg cells [36]. In addition, MSCs can reduce B-cell proliferation through cell–cell contact and secretion of soluble factors [31]. Intravenous injection of MSCs alleviates acute hepatitis and NKT cell hepatotoxicity in an IDO-dependent paracrine manner; however, MSCs did not distinctly alter the total number of neutrophils producing IL-17, CD4^+^, and CD8^+^ T lymphocytes in the injured liver [32]. Furthermore, MSC transplantation can effectively ameliorate liver injury in ALF rats by reducing the number and activity of neutrophils in both peripheral blood and the liver [37].

### The application of MSC-derived conditioned medium

Recently, MSC-conditioned medium (MSC-CM) reportedly had similar therapeutic effects on the treatment of liver failure with MSC transplantation, and the therapeutic effects of MSC-CM may be the combined effect of free soluble factors and exosomes because both of them have been proven to be effective in treating liver failure. Lotfinia et al. reported that MSC-CM can improve liver function but not increase survival rate. In their study, MSC-CM could significantly enhance the viability of primary hepatocytes and increase the secretion of anti-inflammatory IL-10 from human blood mononuclear cells. Meanwhile, the biochemical and histopathological parameters of liver injury were improved 48 h after injection of MSC-CM; however, the survival rate of ALF mice was not increased 1 week after injection [38]. However, many studies have reported that MSC-CM could provide a significant survival benefit in FHF and promote the repair of damaged liver tissue by inhibiting apoptosis in hepatocytes, improving liver regeneration, and reducing panlobular leukocytic infiltrates [22, 36, 39–41]. MSC-CM contains numerous soluble factors associated with the survival benefits of FHF [12, 22, 40, 42]. MSC-CM treatment significantly reduced serum interferon-gamma (IFN-γ), IL-1β, and IL-6 levels and elevated serum IL-10 levels compared with the control group. Proteomic analysis of MSC-CM showed that IL-10 levels increased most significantly in anti-inflammatory factors. Phosphorylation of signal transducer and activator of transcription 3 (STAT3) was upregulated after IL-10 infusion and AG490-induced STAT3 inhibition reversed the therapeutic effects of IL-10 [42]. Moreover, MSC-CM with high levels of HGF and vascular endothelial growth factor can improve the survival rate of ALF rats [22]. Previous studies indicated that conditioned medium derived from different cells has diverse therapeutic effects in liver failure. Conditioned medium of hepatic progenitor-like cells (HPL-CM) is more effective than conditioned medium from AF-MSCs in treating liver failure. Proteomic analysis showed that HPL-CM contained anti-inflammatory factors, including IL-10, IL-1–receptor antagonist (IL-1ra), IL-13, and IL-27, which could induce liver recovery [12]. Huang et al. observed that MSCs had a better therapeutic effect in FHF than MSC-CM by reducing macrophage infiltration into the damaged liver. In contrast, MSC-CM had a better inhibitory effect on fibrogenesis and necroinflammation in chronic liver injury by inhibiting the activation of hepatic stellate cells, promoting liver regeneration, and reducing hepatocyte apoptosis [36].

Notably, conditioned medium can also play a therapeutic role in liver failure via exosomes. Chen et al. found that treatment with menstrual blood stem cell–derived exosomes (MenSC-Ex) before D-GalN/LPS injection could reduce TNF-α, IL-6, and IL-1β levels in circulation of FHF mice, inhibit hepatocyte apoptosis, improve liver function, and ultimately reduce the mortality of FHF mice [43]. Furthermore, MSCs can induce hepatocytes to transform into progenitor oval cells through secretory exosomes. The progenitor oval cells supplement hepatocytes in liver regeneration [44]. It is controversial whether MSC-derived exosomes (MSC-Ex) play a role in the treatment of liver failure through oxidative stress [45, 46]. Yan et al. indicated that MSC-Ex had an anti-oxidant effect; they administered MSC-Ex up to 16 mg/kg body weight through the tail vein to treat liver failure via anti-oxidation and anti-apoptosis. MSC-Ex provided hepatoprotection via anti-oxidation to reduce hepatocyte injury caused by CCl_4_ and hydrogen peroxide (H_2_O_2_) *in vitro* and *in vivo* and this process may be mediated by release of glutathione peroxidase-1 (GPX1) to reduce hepatic reactive oxygen species (ROS) and inhibit oxidative stress–induced apoptosis by upregulating ERK1/2 and Bcl-2 and suppressing the IKKB/NFκB/casp-9/-3 pathway [45]. In contrast, another study showed that MSC-Ex inhibited acetaminophen (APAP) and H_2_O_2_ induced hepatocytes apoptosis by upregulating Bcl-_XL_ protein and promoting the proliferation of hepatocytes. However, MSC-Ex cannot alleviate hepatocyte injury by regulating oxidative stress [46].

### Preclinical studies on MSC therapy for liver failure

Many previous studies have shown that MSC transplantation can improve liver function, inhibit hepatocyte apoptosis, and promote hepatocyte proliferation in animal models of ALF [19, 36, 47–51]. Cai et al. established that BM-MSC transplantation decreased ALT and AST levels, downregulated Bax protein, and increased Bcl-2 expression compared with an acute liver injury (ALI) model [49]. In addition, MSC transplantation in rats can regulate liver and blood metabolic disorders, such as the imbalance of amino acids, bile acids, sphingolipids, acylcarnitines, and glycerophospholipids, which would increase proliferation and decrease apoptosis in hepatocytes [48, 52]. Salomone et al. showed that AT-MSC transplantation in rats with ALI decreased AST, ALT, and prothrombin time (PT) and reduced liver isoprostanes, 8-hydroxyguanosine, and nitrite-nitrate levels but maintained glutathione levels. TNF-α, MCP-1, IL-1β, ICAM-1, and phospho-JNK levels in liver tissue after AT-MSC therapy did not increase significantly [51]. Meanwhile, AT-MSC transplantation remarkably improved the survival of ALF mice and reduced the severity of APAP-induced liver injury by inhibiting cytochrome P450 activity to decrease the accumulation of toxic nitrotyrosine and upregulation of NF-E2–related factor 2, resulting in an increase in anti-oxidant activity. These effects protected hepatocytes against APAP-induced injury by inhibiting the activation of MAPK signaling pathways and the production of inflammatory cytokines [53]. It has been reported that BM-MSCs play a therapeutic role in the pathogenesis of FHF and chronic fibrosis in mice by acting on various cells such as stimulating proliferation and inhibiting apoptosis of hepatocytes, reducing infiltrating macrophages, transforming CD4^+^ T lymphocytes into an anti-inflammatory state, and causing death of hepatic stellate cells [36]. Furthermore, BM-MSCs suppressed ConA-induced inflammatory responses to relieve liver damage by downregulating TNF-a, IFN-γ, and FasL and upregulating IL-10 mRNA [54]. IL-10 has the potential to treat ALF and exert an anti-inflammatory effect through activation of STAT3 signaling pathway and reducing NLRP3-caspase-1 inflammasome levels [42, 55]. In another study, BM-MSC transplantation significantly increased the survival time of pigs with FHF. The treatment group displayed a survival time longer than 14 d compared with the average survival time of 3.22 d in the control group. Analysis of cytokine arrays and metabolite profiles indicated that BM-MSC transplantation inhibited the life-threatening cytokine storm induced by D-GalN and stabilized FHF in pigs within 7 d. Meanwhile, Delta-like ligand 4 was proven to support liver restoration in a pig FHF model [56]. Liu et al. proved that the therapeutic effect of intravenously injected UC-MSCs on reducing hepatocyte apoptosis and enhancing liver regeneration was mediated by paracrine pathways, involving the reduction of anti-oxidants (glutathione and superoxide dismutase), inflammatory factors (TNF-α and IL-6), and the increase of serum HGF levels [57]. Tonsil-derived MSCs (T-MSCs) express galectin-1, -3, -8, and -9; however, expression of galectin-1 and -3 is more prominent than that of other galectins. Galectin-1 knockout reduces the immunosuppressive effect of T-MSCs on CD4^+^ T cells [58]. After galectin-1 knockout, CD4^+^ and CD8^+^ T-cell proliferation recovered partially. However, the effect of MSCs on NK cells was unaffected by the downregulation of galectin-1. MSC-derived galectin-1 significantly regulated the release of cytokines, including TNF-α, IFN-γ, IL-2, and IL-10 [59]. In D-Gal/LPS–induced ALF rats, BM-MSCs could significantly inhibit the nuclear factor-kappa B (NF-κB) pathway and reduce the levels of inflammatory factors, including IL-1β, IL-6, and TNF-α, by upregulating heme oxygenase-1 (HO-1) [60]. Zheng et al. found that the high-mobility group box 1 protein (HMGB1) in serum and liver tissues is positively associated with liver damage. BM-MSC transplantation can improve liver function and liver pathology in ALF rats and reduce serum and liver HMGB1 [61]. Taken together, these preclinical studies clearly demonstrate that MSCs can effectively treat liver failure and explain the potential treatment mechanisms.

### Clinical trials of MSCs in the treatment of liver failure

At present, although there are fewer clinical studies on MSC therapy for liver failure, previous studies have consistently agreed that MSCs can effectively treat liver failure. In an open-label randomized controlled study, allogeneic BM-MSC transplantation reduced serum TBIL and model for end-stage liver disease (MELD) scores in 56 patients with hepatitis B virus (HBV)-related acute-on-chronic liver failure (ACLF) that were injected with about 1 to 10 × 10^5^ cells/kg weekly for 4 weeks and followed up for 24 weeks. Meanwhile, BM-MSCs can reduce the incidence of serious infection and increase the cumulative survival rate. After treatment with BM-MSCs, no tumors were detected in any trial patients; however, fever occurred more frequently [62]. In a 24-month prospective study, the researchers used a single infusion of 100 × 10^6^ UC-MSCs through the hepatic artery to treat 11 patients with HBV-related ACLF. In the treatment group, serum ALB, ALT, AST, bilirubin, direct bilirubin, PT, international standardized ratio (INR), and MELD scores were significantly improved after 4 weeks of UC-MSC transplantation, and levels of ALB, PT, and INR also increased significantly at 24 months [63]. In a clinical trial conducted by Shi et al., UC-MSCs (0.5 × 10^6^ cells/kg) were intravenously infused three times at 4-week intervals to assess their therapeutic effects in 20 patients with HBV-associated ACLF. UC-MSC infusion significantly increased serum ALB, cholinesterase, prothrombin activity, platelet counts, and survival rate of patients with ACLF and decreased TBIL, ALT levels, and MELD scores [64]. Autologous BM-MSC transplantation through the hepatic artery is safe for patients with HBV-associated liver failure. Short-term outcomes are favorable; however, long-term outcomes have not improved significantly. The levels of ALB, TBIL, PT, and MELD scores in the transplantation group were significantly improved at about 2 to 3 weeks after transplantation; however, incidence of hepatocellular carcinoma or mortality did not differ significantly between the BM-MSC transplantation group and the control group after 192 weeks of follow-up [65].

### Exploring the best method of MSC therapy for liver failure

Although many studies have confirmed the effectiveness of MSCs in treating liver failure, there is no standard protocol for MSC therapy. Numerous issues need further investigation regarding MSC treatment for liver failure, including selection of the optimal transplantation route, therapeutic effects of MSCs from different sources, and MSC colonization *in vivo*.

The principal methods involved in MSC transplantation include the peripheral, portal, and splenic veins; hepatic artery; intrahepatic injection; and intrasplenic injection, which may have different therapeutic effects in liver failure. Several studies suggested that administration via the portal vein had better results compared with other transplantation routes. Portal vein injection was superior to other MSC transplantation methods, including the hepatic artery, peripheral vein, and intrahepatic injection. Intraportal MSC injection could improve liver function, inhibit cell apoptosis, and prolong the survival time of pigs with ALF [66]. Similarly, MSCs via portal vein transplantation had better capability to reduce liver inflammation, decrease liver degeneration and necrosis, and promote liver regeneration compared with peripheral vein administration [67]. However, another study suggested that the liver function of rats with liver failure was significantly improved after MSC transplantation via three different routes, namely the hepatic artery, portal vein, and peripheral vein, but no significant difference was observed among the three groups [68]. There was no significant difference in liver enzyme, albumin, bilirubin, and hemoglobin levels; total white blood cell count; and platelets in patients with liver failure treated via intrahepatic and intrasplenic injection of MSCs [47, 69]. In addition, UC-MSC transplantation through the peripheral vein displayed similar curative effects with intrahepatic injection [19]. After transplantation of AT-MSCs through the peripheral vein and the splenic vein, ALF was improved in pigs. Splenic vein transplantation has better therapeutic effects than peripheral vein transplantation in protecting liver function, reducing pro-inflammatory factors, increasing anti-inflammatory factors, and promoting liver regeneration [70].

Hepatic colonization of MSCs is one of the principal factors affecting MSC therapy for liver failure. Zhu et al. showed that BM-MSCs were detected in injured liver tissue at 24 h after transplantation, and no BM-MSCs were detected in mice without liver injury, suggesting that tissue injury could recruit BM-MSCs *in vivo*. All transplanted BM-MSCs located near the hepatic portal vein disappeared [33]. Furthermore, MSCs transplanted into the liver were related to the presence of liver injury [20, 71] and unrelated to the injection site [18, 72]. In the current MSC transplantation, only a few MSCs can colonize the liver, and we can improve the hepatic colonizability by modifying MSCs. c-Met-MSCs significantly enhanced the homing ability to the injured liver and increased the survival rate and liver function in rats with liver failure [73]. A similar study demonstrated that MSCs expressing C-X-C chemokine receptor type 4 (CXCR4) had greater hepatic colonizability and contributed to restore the damaged liver [74, 75]. It has been reported that stromal cell–derived factor-1 (SDF-1), as a chemotactic factor, may promote the migration of MSCs to the liver through the SDF-1/CXCR4 axis and that SDF-1 mobilizing MSCs can enhance liver regeneration after liver injury [76].

It is unclear whether MSCs from different sources have the same therapeutic effects on liver failure. Compared with the results of biochemical analysis, histopathological assessment, gene expression of HLCs, and survival rate, AT-MSCs treated liver failure more effectively than BM-MSCs did [24, 77]. However, another study established that only BM-MSCs reduced liver damage and alleviated liver failure in ConA-induced mice, compared with mature hepatocytes, fetal liver cells, and induced hepatic stem cells [54]. Recently, it has been suggested that combined transplantation of MSCs and mature hepatocytes in ALF may be a good combination to facilitate liver repair and anti-inflammatory effects [78, 79]. The liver failure microenvironment contributed to the transplanted MSCs to express hepatocyte-specific genes [80, 81]. Meanwhile, ALF upregulated liver-specific genes in MSCs but did not affect its stem cell characteristics and cell viability [80]. The serum from rats with ALF induced the expression of CXCR4 on MSCs, which enhanced the homing ability of MSCs to damaged liver tissue [75].

In addition to transplantation of MSCs alone, liver failure may be synergistically treated by enhancing growth-related gene expression or blocking the effect of inflammatory factors. Tang et al. reported that UC-MSCs overexpressing HGF can reduce liver damage and prolong the survival rate of APAP-induced ALF mice through anti-apoptosis and anti-oxidation [82]. Wang et al. found that c-Met expression in hepatocytes is closely associated with HGF-mediated liver regeneration [73]. Hepatocytes could specifically express the c-Met gene through gene transfer *in vivo*, thus enhancing hepatocyte proliferation, decreasing apoptosis, and significantly improving overall survival rates [83]. It has been shown that IL-1Ra is a natural IL-1 antagonist, which can block the inflammatory process through competitive binding with IL-1 receptor [84]. Therefore, IL-1Ra could decrease IL-1, IL-6, TNF-α, and other inflammatory markers in a short period [21, 85]. MSCs overexpressing IL-1Ra gene can be transplanted into the injured liver to improve liver function and survival rate in animal models of ALF [85]. Similar results showed that combined treatment of IL-1Ra and MSCs could promote the recovery in pigs with ALI and had better therapeutic effects than MSCs alone [86, 87].

## Conclusion

MSCs have many properties, including immunoregulation, differentiation into HLCs, and repair of damaged tissue, which contribute to the treatment of liver failure. Many previous studies have shown that MSCs can effectively treat liver failure, most preclinical studies but few clinical studies. In most of the existing studies, researchers observed the short-term benefits of MSC therapy but long-term efficacy was lacking. Therefore, it is unclear whether MSC therapy can provide long-term benefits. The mechanism of MSC treatment for liver failure is primarily focused on differentiation of MSCs into hepatocytes and immunoregulation, and the effect of immunoregulation seems more obvious. In addition to MSC therapy alone, MSC modification or MSCs combined with other treatment methods are being increasingly considered. But there are still many problems to be solved in standardizing the process of MSC therapy for liver failure, including determination of the optimal time, dose, and route for MSC transplantation; improvement of the colonization rate and survival rate of MSCs in the liver; and the safety of MSC transplantation. Consequently, although MSCs have great potential in treating liver failure, they are facing numerous challenges prior to clinical application.

## Abbreviations

ACLF: Acute-on-chronic liver failure
AF-MSC: Amniotic fluid mesenchymal stem cell
ALB: Albumin
ALF: Acute liver failure
ALI: Acute liver injury
ALT: Alanine transaminase
APAP: Acetaminophen
AST: Aspartate transaminase
AT-MSC: Adipose tissue mesenchymal stem cell
bFGF: Basic fibroblast growth factor
BM-MSC: Bone marrow mesenchymal stem cell
CCl_4_: Carbon tetrachloride
CXCR4: C-X-C chemokine receptor type 4
D-GalN/LPS: D-galactosamine/lipopolysaccharide
EGF: Epidermal growth factor
FasL: Fas ligand
FHF: Fulminant hepatic failure
H_2_O_2_: Hydrogen peroxide
HBV: Hepatitis B virus
HGF: Hepatocyte growth factor
HLC: Hepatocyte-like cell
HMGB1: High-mobility group box 1
HPL-CM: Conditioned medium of hepatic progenitor-like cells
IDO: Indoleamine 2,3-oxidase
IFN-γ: Interferon-gamma
IL: Interleukin
IL-1Ra: Interleukin-1–receptor antagonist
INR: International standardized ratio
ITS: Insulin transferrin-selenium
MELD: Model for end-stage liver disease
MSC: Mesenchymal stem cell
MSC-CM: Mesenchymal stem cell conditioned medium
MSC-Ex: Mesenchymal stem cell–derived exosomes
NK: Natural killer
OSM: Oncostatin M
PT: Prothrombin time
SDF-1: Stromal cell–derived factor-1
STAT3: Signal transducer and activator of transcription 3
TBIL: Total bilirubin
TGF-β: Transforming growth factor-beta
Th: T helper
T-MSC: Tonsil-derived mesenchymal stem cell
TNF-α: Tumor necrosis factor-alpha
Treg: Regulatory T
UC-MSC: Umbilical cord mesenchymal stem cell
